# The association between deaths from infection and mutations of the *BRAF, FBXW7, NRAS and XPO1* genes: a report from the LRF CLL4 trial

**DOI:** 10.1038/s41375-021-01165-w

**Published:** 2021-02-12

**Authors:** Monica Else, Stuart J. Blakemore, Jonathan C. Strefford, Daniel Catovsky

**Affiliations:** 1 Division of Molecular Pathology, The Institute of Cancer Research, London, UK; 2 Cancer Genomics, School of Cancer Sciences, Faculty of Medicine, University of Southampton, Southampton, UK; 3 Department I of Internal Medicine, University Hospital Cologne, Cologne, Germany

**Keywords:** Chronic lymphocytic leukaemia, Cancer genetics, Infectious diseases

## Abstract

Causes of death, in particular deaths due to infection, have not been widely studied in randomised trials in chronic lymphocytic leukaemia. With long-term follow-up (median 13 years) we examined the cause of death in 600/777 patients in the LRF CLL4 trial. Blood samples, taken at randomisation from 499 patients, were available for identifying gene mutations. Infection was a cause of death in 258 patients (43%). Patients dying of infection were more likely than those who died of other causes to have received ≥2 lines of treatment (194/258 [75%] versus 231/342 [68%], *P* = 0.04) and to have died in the winter months (149/258 [58%] versus 166/342 [49%], *P* = 0.03), respectively. In patients with mutation data, the factors significantly associated with death from infection versus all other deaths were 11q deletion (47/162 [29%] versus 40/209 [19%], *P* = 0.03) and mutations of the *BRAF, FBXW7, NRAS* and *XPO1* genes. Death was caused by an infection in 46/67 assessable patients (69%) who had a mutation of one or more of these four genes versus only 129/333 patients (39%) without any of these mutations (odds ratio: 3.46 [95% CI 1.98–6.07] *P* < 0.0001). Careful management of infection risk, including prophylaxis against infection, may be important in patients who carry these mutations.

## Introduction

Although overall survival (OS) has been an endpoint in many clinical trials in chronic lymphocytic leukaemia (CLL), the specific causes of patients’ deaths have not been widely studied in randomised trials. The LRF CLL4 trial is uniquely appropriate for this investigation for two reasons. First, it has long-term follow-up (median 13 years), which allowed us to examine the cause of death in 600 of the 777 patients randomised. Secondly, blood samples taken at randomisation from 499 patients are still available today for an analysis of baseline molecular data. Thus we have been able to examine the relationship between deaths from infection and a large panel of genes which have only relatively recently been found to be commonly mutated in CLL.

Infections are the leading cause of death in CLL, with around half of patients succumbing [1–3]. A study of 10,455 patients with CLL from the Danish Cancer Registry demonstrated a significant improvement in OS between 1978 and 2013, in parallel with the introduction of chemo-immunotherapeutic agents, but the risk of death from infections in CLL patients did not change over this time period. It remained 50% higher than for a matched cohort of 508,995 individuals without CLL [4]. Meanwhile, the organisms causing infections have changed with the introduction of purine analogues and monoclonal antibodies. Formerly mostly common bacterial organisms, they now include less common opportunistic pathogens such as listeria, candida, aspergillus, and pneumocystis jirovecii [2, 5, 6]. It has been suggested that the newer tyrosine kinase inhibitor ibrutinib might reduce the rate of infections [7–9].

The reasons why patients with CLL are susceptible to infections are multifactorial, including both therapy-related immunosuppression and defects of cellular and humoral immunity which are facets of the disease itself and which tend to increase over the course of the disease [3, 5, 10]. Factors may include defective T-cell and B-cell function, with low levels of serum immunoglobulins (hypogammaglobulinaemia) and defects in complement activity and neutrophil/monocyte function, all contributing to profound immune dysregulation [1, 2, 5, 6, 10, 11]. CLL treatment trials have identified various risk factors associated with the incidence of infections. These factors differ according to the treatments given and the patient demographics, but they include two or more previous lines of therapy, advanced disease stage, elevated LDH, low haemoglobin, low baseline IgG and IgA levels, renal insufficiency and older age [1, 5, 6, 8]. The Danish group have also identified factors associated with the severe infection prior to treatment in patients in the Danish National CLL Registry, notably an elevated β2 microglobulin level, low levels of IgA and a shorter time between previous infections [8, 12]. To our knowledge, risk factors in CLL for infections which result in death have not been separately studied, except recently in the setting of Covid-19 [13, 14].

Advances in identifying genes commonly mutated in CLL have led to the identification of several mutations which are linked to an earlier death. In the LRF CLL4 trial mutations of *TP53* [15], *SF3B1* [16], *NOTCH1* (coding [16] and non-coding [17]) *ATM* with 11q deletion [18], *EGR2* [19], and biallelic *BIRC3* as well as *MAPK-ERK* genes [20] have all been shown to be predictive of shorter OS. In this study, we aimed to identify gene mutations which were specifically associated with death caused by infection.

## Subjects and methods

LRF CLL4 was a large multi-centre randomised trial of 777 patients with previously untreated CLL. Patients were randomised between 1999 and 2004 to receive chlorambucil or fludarabine, with or without cyclophosphamide. Clinical follow-up was to October 2010 (median 7.1 years; range 6.0–11.7 years). Follow-up for OS was censored at October 2010 for 44 patients who were resident outside the UK but continued until September 2016 for UK-based patients (median 13.3 years follow-up for surviving patients; range 11.9–17.6 years). Causes of death were assessed centrally by the principal investigator, Daniel Catovsky, in his capacity as the single reviewer of all the death certificates and clinicians’ reports. For each patient up to two main causes of death were identified and classed simply as: CLL; Richter’s syndrome; infection; vascular; other cancer; other unclassified cause. Then, for this study, all the patients who had infection listed as one of their two causes of death were categorised as having died of infection. Thus this category did *not* include patients with incidental infections which were present at the time of death but which were not considered to be a main cause of death. For our primary analysis, the patients who died of infection were contrasted with all the other patients who died, i.e., those whose categorised causes of death did not include infection. A secondary analysis compared these same two groups within the subset of patients with available gene mutation data.

The following demographic, laboratory and molecular characteristics were recorded at randomisation, or assessed in stored blood samples collected at randomisation: age, sex, disease stage, size of the treatment centre, randomised treatment, haemoglobin and platelet levels, white blood count, lymphocyte and prolymphocyte counts, beta-2 microglobulin and lactate dehydrogenase levels, lymphadenopathy, *IGHV* mutation status, CD38 and Zap70 expression, telomere length, presence of trisomy 12 and deletions of chromosomes 13q, 11q and 17p. The cut-offs used for these variables have been previously published [21]. Not all the variables were assessable in all patients. Data on 623 high-confidence somatic mutations in *ATM, BIRC3, BRAF, CHD2, DDX3X, EGR2, FBXW7, KRAS, MED12, MGA, MYD88, NFKBIE, NOTCH1, NRAS, POT1, RPS15, SAMHD1, SETD2, SF3B1, TP53* and *XPO1* were available for analysis from our previous work, in 499 patients [20]. Cut-offs to define summer versus winter deaths were: 15th April (start of summer) and 15th October (start of winter)—reversed for patients in the southern hemisphere. World Health Organisation performance status (0 versus 1–4) was measured 6 months after the start of first-line treatment. The number of lines of treatment received and responses to treatments were also considered.

### Statistics

Generalised linear modelling was used to identify which of the above variables were associated with deaths from infection versus patients whose causes of death did not include infection. Values of *P* ≤ 0.05 (two-sided) were considered significant and were included in a multivariate model using forward stepwise analysis. The Kaplan–Meier method was used to estimate OS. STATISTICA software from StatSoft, a subsidiary of Dell, Inc. (Tulsa, OK, USA) was used for the analyses. In addition, for the 21 genes with available data, Fisher’s exact test was used in R v3.3.0, followed up with multiple hypothesis testing.

LRF CLL4 is registered as an International Standard Randomised Controlled Trial, number NCT 58585610 and was approved by the UK multicentre research ethics committee. Informed consent was obtained from all subjects.

## Results

**Fig. 1:**
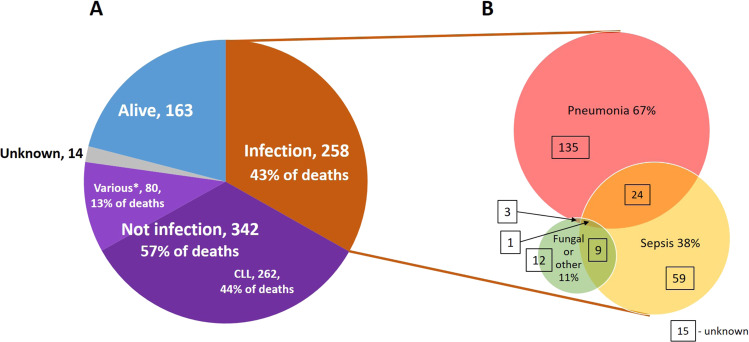
Mortality in the LRF CLL4 trial (*n* = 777). **A** Causes of death. *‘Various’ included 21 cardiovascular causes and 46 other cancers. **B** Fatal infections. *Note*: this is a diagrammatic representation of the data. The areas of the circles correspond to the number of patients in each category, but the areas of the overlapping sections do not.

In the LRF CLL4 trial, 614 of 777 patients (79%) died before the end of follow-up. The cause of death was known in 600 patients. Deaths tended to have more than one cause, but CLL was a cause in at least 520/600 patients (87%), including 258 deaths (43%) from infection (Fig. 1A).

Fatal infections were pneumonia (67%), and/or sepsis (38%) and/or opportunistic infections (11%) (Fig. 1B). The latter group consisted of fungal infections (*n* = 9, 4%), mainly aspergillus, plus a range of other infections (*n* = 16, 7%) including adenoviral enteritis, clostridium difficile colitis, cryptosporidium colitis, cytomegalovirus, encephalitis, hepatitis, klebsiella, legionella pneumonia, listeria, necrotising fasciitis, parainfluenza and tuberculosis.

Of 137 patients who died from infection before the end of clinical follow-up in 2010, 59 (43%) died within 6 calendar months of the start of a line of treatment. Thus 13 died during first-line, 31 during second-line and 15 during third-line treatment. The remaining 57% died of infection when they were off treatment.

### Comparing patients who died from infection versus those who died of all other causes

In the full dataset of 600 patients whose cause of death was known, the patients who died from infection were more likely than those whose death was not due to infection to have received two or more lines of treatment (Table 1A). Deaths from infection were more likely to occur during the winter, while deaths not due to infectious causes were evenly spread between the summer and winter months. Among the panel of 21 genes commonly mutated in CLL, univariate analysis showed that mutations of *BRAF, FBXW7, NRAS* and *XPO1* were significantly associated with death from infection versus deaths not due to infection. However, *NRAS* was the only one of these recurrently mutated genes to remain significantly associated with death from infection after Benjamini and Hochberg false discovery adjustment (FDR, *Q* > *P* [*P* < 0.05]) (odds ratio: 17.51, 95% confidence interval (CI): 2.20–139.40, *P* = 0.0004); (data not shown). No other significant differences were found between patients who died of infection versus those who died from other causes, with respect to any other of the demographic, disease or treatment characteristics listed in the “Subjects and methods”. In particular, the rate of deaths from infection was not influenced by disease stage, the randomised treatment, the response to treatment, or the size/experience of the CLL treatment centre, as measured by the number of patients entered in the trial.

**Table 1:** Univariate and multivariate analyses of baseline variables associated with deaths from infection versus deaths not due to infection.

Variable	Deaths from infection (%)	Deaths not due to infection (%)	Odds ratio	95% confidence levels	*P*-value
A. Univariate analysis: all trial patients with a known cause of death (*n* = 600)					
≥2 lines of treatment	194/258 (75)	231/342 (68)	1.46	1.01–2.09	0.04
Winter season	149/258 (58)	166/342 (49)	1.45	1.05–2.01	0.03
*BRAF* mutation	19/175 (11)	9/225 (4)	2.92	1.29–6.63	0.01
*FBXW7* mutation	8/175 (5)	2/225 (1)	5.34	1.12–25.48	0.04
*NRAS* mutation	9/175 (5)	1/225 (0.4)	12.14	1.52–96.79	0.02
*XPO1* mutation	17/175 (10)	9/225 (4)	2.58	1.12–5.94	0.03
B. Univariate analysis: patients with a known cause of death and gene mutation data (*n* = 400)					
11q deletion	47/162 (29)	40/209 (19)	1.73	1.06–2.80	0.03
*BRAF* mutation	19/175 (11)	9/225 (4)	2.92	1.29–6.63	0.01
*FBXW7* mutation	8/175 (5)	2/225 (1)	5.34	1.12–25.48	0.04
*NRAS* mutation	9/175 (5)	1/225 (0.4)	12.14	1.52–96.79	0.02
*XPO1* mutation	17/175 (10)	9/225 (4)	2.58	1.12–5.94	0.03
C. Multivariate analysis: patients with a known cause of death and gene mutation data (*n* = 400)					
11q deletion			1.79	1.09–2.94	0.02
*BRAF* mutation			3.22	1.33–7.80	0.01
*NRAS* mutation			10.78	1.31–88.43	0.03
*XPO1* mutation			2.49	1.01–6.12	0.05

Of the 499 patients in the trial for whom gene mutation data were available, 411 died. Of these, 400 had a known cause of death. In the univariate analysis in this subset of 400 patients, 11q deletion was significantly associated with death from infection versus deaths not due to infection, along with mutations of the *BRAF, FBXW7, NRAS* and *XPO1* genes (Table 1B). Multivariate analysis in these 400 patients showed that the factors most significantly associated with death from infection were 11q deletion and mutations of the *BRAF, NRAS* and *XPO1* genes (Table 1C).

**Fig. 2:**
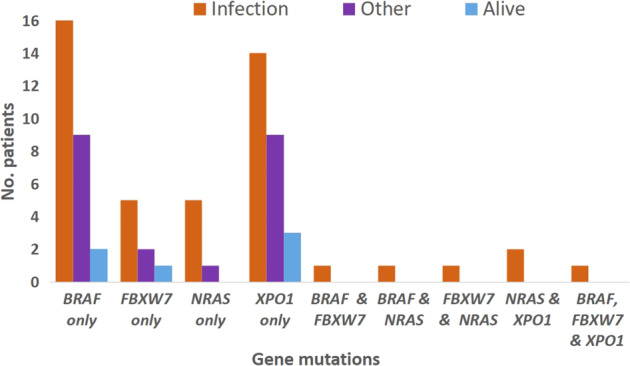
Incidence of *BRAF, FBXW7, NRAS* and *XPO1* mutations in deaths from infection, other deaths and patients remaining alive (*n* = 73). 73/499 patients with gene mutation data carried one or more of these mutations.

Of the 499 patients with gene mutation data, 73 (15%) carried one or more of the four gene mutations *BRAF* (6%)*, FBXW7* (2%), *NRAS* (2%) and *XPO1* (6%). Of these, 67 (92%) died and only six (8%) remained alive (Fig. 2). Death was caused by an infection in 46 of the 67 who died (69%). In contrast, in the patients who died but did not carry any of the four mutations, an infection was a cause of death in only 129/333 (39%). Alternatively, of those who died of infection 46/175 assessable patients (26%) carried one of these four mutations versus only 21/225 (9%) of those who died of causes which did not include infection. (Odds ratio: 3.46 [95% CI: 1.98–6.07] *P* < 0.0001).

### Details of the patients with BRAF, FBXW7, NRAS and/or XPO1 mutations

Of the 30 *BRAF*-mutated patients in the trial 19 (63%) died of infection, nine of other causes and two survived (Fig. 2). Eleven patients had an *FBXW7* mutation of whom eight (73%) died of infection, two died of other causes and one survived. There were ten *NRAS*-mutated patients of whom nine (90%) died of infection and one died of another cause. The single patient with an *NRAS* mutation who did not die of an infection nevertheless had an episode of severe and prolonged neutropenic sepsis after her first course of chlorambucil, followed by pneumocystis pneumonia 3 years later after completing second-line treatment with fludarabine. Of 29 trial patients with an *XPO1* mutation, 17 (59%) died of infection, nine died of other causes and three survived. All six of the patients who carried more than one of these mutations died of infection.

The types of fatal infection were similar in these 46 patients to those for the trial as a whole (Fig. 1B), totalling 34 cases of pneumonia, 13 of sepsis, two of other infections and two with unspecified infections.

The demographics, clinical characteristics, and prognostic biomarkers of patients with versus without *BRAF, FBXW7, NRAS*, and/or *XPO1* mutations are shown in Table 2. Patients with one or more of these mutations were significantly more likely than other patients to have unmutated *IGHV* genes, positive CD38 and ZAP70 expression, trisomy 12, absence of 13q deletion and a *KRAS* and/or *NOTCH1* mutation. They were significantly less likely than others to have an *SF3B1* mutation.

**Table 2:** Demographics, clinical characteristics, and prognostic biomarkers in patients with versus without *BRAF, FBXW7, NRAS*, and/or *XPO1* mutations (*n* = 499^a^).

Patient/disease characteristic	*BRAF, FBXW7, NRAS*, and/or *XPO1* mutation		*P*
	Yes % (*n* = 73)	No % (*n* = 426)	
Aged >70 years	34	30	0.5
Male	78	71	0.2
Binet stage C^b^	36	31	0.4
Randomised arm chlorambucil	52	47	0.5
Non-response to treatment	21	21	1.0
≥2 lines of treatment	73	66	0.3
Unmutated *IGHV* genes (>98% homology)^a^	84	59	0.0002
β2 microglobulin ≥4 mg/L^a^	55	47	0.3
CD38 positive (cut-off 7%)^a^	78	61	0.02
Zap-70 positive (cut-off 10%)^a^	65	47	0.02
Trisomy 12^a^	29	14	0.003
11q deletion^a^	24	20	0.5
Absence of 13q deletion^a^	62	37	0.0001
17p deletion^a^	8	5	0.5
*ATM* mutation	8	7	0.8
*BIRC3* mutation	12	6	0.07
*EGR2* mutation	1	3	0.5
*KRAS* mutation	15	4	0.0003
*NOTCH1* mutation	21	11	0.02
*SF3B1* mutation	15	26	0.05
*TP53* mutation	15	9	0.09

^a^Eight variables had missing data, with the no. of cases ranging from 356 to 464.

^b^Binet stage C disease was not significant because, while patients with these gene mutations were more likely than others to have anaemia (haemoglobin < 100 g/L; 33% versus 18%; *P* = 0.003), they were less likely to have thrombocytopenia (platelets < 100 × 10^9^/L; 10% versus 21%; *P* = 0.02).

### Overall survival

Median OS from randomisation for the whole trial was 6 years, with surviving patients censored at their last follow-up date. OS was the same for patients who died of infection as it was for those who died of other causes: median 4 years 6 months versus 4 years 5 months, respectively. For the 67 patients carrying just one of the four mutations, there was no significant difference in OS between those who died from infection and those whose deaths were not due to infectious causes (median 4 years 1 month and 4 years 5 months, respectively). The median OS of the six patients who had more than one of the four mutations was 2 years 2 months.

## Discussion

By 13 years median follow-up 79% of the patients in LRF CLL4 had died, the majority due to CLL or its complications, including infection, which was a cause in 43% of deaths. We do not have information on the particular pathogens involved, but two-thirds of deaths from infection had a respiratory origin.

Well-known adverse prognostic factors, associated with shorter OS in CLL, such as older age, male sex, *TP53* or *NOTCH1* abnormalities, or unmutated *IGHV* genes, were not significantly different between patients who died from infection and those who died from other causes. Many of these factors are also associated with poor response to treatment, as previously reported [21], but in this study patient who failed to respond to treatment succumbed to their CLL, either with or without infection in equal measure. Although stage C disease has been associated with a greater likelihood of severe infections [8], in this trial patients with stage C disease at randomisation were at no greater risk of death from infection than patients with stage A-progressive or stage B disease. Deletion of 11q was associated with death from infection in the subset of patients with mutation data, though not in the full trial dataset.

CLL is characterised by progressive immunodeficiency [2, 3, 8]. We found that patients who had undergone two or more lines of treatment were at greater risk of death from infection, consistent with previous reports associating longer disease duration with a greater likelihood of severe infections [5, 6, 22, 23], especially in heavily pre-treated patients with active disease [10]. In CLL severe infections are already common prior to treatment, with a five-year cumulative incidence of 31% among 2905 CLL patients [8], while documented infections may even predate CLL diagnosis by years, or decades, increasing in incidence over time [24]. This latter finding suggests that infections might contribute to CLL development through antigen stimulation, inducing somatic mutations, or epigenetic modifications in line with the antigenic drive in lymphoid malignancies [24]. Several of the gene polymorphisms implicated in CLL risk by GWAS suggest a key role for aberrant immune responses in CLL pathogenesis, suggesting that cumulative immune dysfunction is critical to CLL development [25].

With this in mind, our finding is intriguing but unsurprising that specific gene mutations, identified in samples obtained prior to initial treatment, namely those of the *BRAF, FBXW7, NRAS* and *XPO1* genes, may, after further disease progression, be associated with infections so severe they result in death.

A possible mechanism for this has been reported with respect to *NRAS*, which is an oncogene involved in regulating cell division. When the human *NRAS* gene was activated in vitro, through point mutation in codon 61, in cells which were incubating the delNS1 H5N1 influenza virus, a significantly increased replication of the virus was observed in these cells compared with controls, while at the same time expression of IRF-3 and IFN-beta decreased [26]. The authors concluded that *NRAS* activated through point mutation, appeared to inhibit IFN antivirus defences and afford a more favourable environment for replication of the delNS1 influenza virus. In a similar experiment, Metzger et al. demonstrated that a mutated-*NRAS* recombinant virus was capable of transforming cells [27]. It should be noted that these findings apply to viral infections only and hence cannot be translated to bacterial or fungal infections.

*BRAF* mutations result in deregulation of B-cell receptor (BCR) intracellular signalling leading to disruption of hematopoietic and early B-cell differentiation [28]. *FBXW7* is a potent tumour suppressor which is also involved in maintaining normal haematopoiesis. Mounting evidence has indicated the involvement of aberrant expression of *FBXW7* for tumorigenesis [29]. An *XPO1* mutation is a driving event in B cell malignancies through alteration of the nuclear trafficking of proteins involved in inflammatory signalling, DNA repair, RNA export and chromatin remodelling pathways [30]. Thus these three gene mutations may each be involved in CLL pathogenesis. *BRAF*, like *NRAS*, is a component in the *MAPK/ERK* signalling pathway, which may be usurped by DNA and RNA viruses to mediate multiple aspects of the virus infectious cycle and thus facilitate viral replication and subsequent pathogenesis [31]. But we are not aware of any studies, like that cited above for the *NRAS* gene, which might cast light on the possible mechanisms leading to a susceptibility to fatal infections in patients with a *BRAF* mutation, nor in those with an *FBXW7* or *XPO1* mutation.

The question arises whether patients who die of infection have a different disease course from those who die of other causes. IgA abnormalities at diagnosis have been shown to predict for both subsequent infections and a more aggressive disease course [9]. Another proposal is that infection may be the driver of more aggressive disease through tumour microenvironment interactions [12, 24]. Vendramini et al. showed a strong association between *KRAS/NRAS/BRAF* mutations and the presence of unmutated *IGHV* genes and trisomy 12 [32]. Similarly, we found that unmutated *IGHV* genes and trisomy 12, as well as *KRAS* mutations, were strongly associated with *BRAF/FBXW7/NRAS/XPO1* mutations, suggesting a complex involvement of these mutations in CLL pathogenesis. We have previously shown an association of *BRAF* and *NRAS* mutations with shorter OS in this trial [20]. Our data in the present study (Fig. 2) suggest this may be explained by the tendency of patients with these mutations to succumb to fatal infections. Patients who died of other causes were less likely to present with these mutations.

It is hoped that an understanding of the molecular events underlying the susceptibility to infections in some patients may lead to improvements in infection control and targeted treatments. The main limitations of this study were: (1) the small number of patients with each of these gene mutations; (2) the ending of clinical follow-up in 2010, with the result that we have no data on the treatments received by these patients since then; and (3) the fact that the randomised treatments in LRF CLL4 are no longer in general use, raising the question as to whether deaths from infection may follow a different pattern in the era of BCR and BCL2 inhibitors. Other researchers may want to examine their databases to see if their findings match ours.

In conclusion, patients in LRF CLL4 were at some risk of death from infection, irrespective of their demographic characteristics, disease stage and treatment history. Nevertheless, those who had received two or more lines of treatment were particularly at risk, as were those who carried a *BRAF, FBXW7*, *NRAS* or *XPO1* mutation. A meta-analysis of datasets from other trials could be important to assess the validity of the link between these gene mutations and deaths from infections in patients with CLL. Careful management of infection risk, including appropriate choice of CLL therapeutic agents and prophylaxis against infection, may be important in patients who carry one or more of these mutations.
